# Salidroside inhibited the proliferation of gastric cancer cells through up-regulating tumor suppressor miR-1343-3p and down-regulating MAP3K6/MMP24 signal molecules

**DOI:** 10.1080/15384047.2024.2322206

**Published:** 2024-03-04

**Authors:** Xiaoping Wang, Zhendong Zhang, Xiaolan Cao

**Affiliations:** Department of Medicine, KeyLaboratory of High Altitude Hypoxia Environment and Life Health, Xizang Minzu University, Xianyang, Shaanxi, P.R. China

**Keywords:** Salidroside, gastric cancer cell, microRNA (miRNA), proliferation, signal molecules

## Abstract

Salidroside inhibited the proliferation of cancer cell. Nevertheless, the mechanism has not been completely clarified. The purpose of the study is to explore the mechanisms of salidroside against gastric cancer. To analyze the changes of microRNA (miRNA) in gastric cancer cells under the treatment of salidroside, the miRNA expression was analyzed by using RNA-seq in cancer cells for 24 h after salidroside treatment. The differentially expressed miRNAs were clustered and their target genes were analyzed. Selected miRNA and target mRNA genes were further verified by q-PCR. The expressions of target genes in cancer cells were detected by immunohistochemistry. Cancer cell apoptotic index was significantly increased after salidroside treatment. The proliferation of gastric cancer cells were blocked at S-phase cell cycle. The expression of 44 miRNAs changed differentially after salidroside treatment in cancer cells. Bioinformatic analysis showed that there were 1384 target mRNAs corresponding to the differentially expressed miRNAs. Surprisingly, salidroside significantly up-regulated the expression of tumor suppressor miR-1343-3p, and down-regulated the expression of MAP3K6, STAT3 and MMP24-related genes. Salidroside suppressed the growth of gastric cancer by inducing the cancer cell apoptosis, arresting the cancer cell cycle and down-regulating the related signal transduction pathways. miRNAs are expressed differentially in gastric cancer cells after salidroside treatment, playing important roles in regulating proliferation and metastasis. Salidroside may suppress the growth of gastric cancer by up-regulating the expression of the tumor suppressor miR-1343-3p and down-regulating the expression of MAP3K6 and MMP24 signal molecules.

## Introduction

The incidence rate of gastric cancer is 17.6/10 million worldwide. In China, the incidence rate is 33.14/10 million, and the mortality rate of gastric cancer is 24.34/10 million.^1^ At present, there are lots of chemotherapeutic drugs for gastric cancer, but the remission period is still short, because chemotherapy is prone to bring about drug resistance and serious adverse effects. With the rapid advancement of molecular tumor biology, it is well known that the occurrence, progression and metastasis of cancer is a complicated biological process with various gene participation. Therefore, searching for potential drug target genes has become an important field of current research.

Rhodiola, as a traditional Chinese herb, has many advantages in various illness treatment, especially for cancer therapy. Salidroside is a phenolic metabolite of Rhodiola, whose aglycone is phydroxyphenylethanol, and has attracted much more attention in its extensive pharmacological effects.^2–4^ Salidroside has been widely used in the prevention of aging, depressant, altitude sickness, anti-fatigue and anti-tumor. Salidroside also has neuroprotective activity, enhancing immune function and protecting the body from radiation damage.^4–7^ It has been proved that salidroside possesses a definite inhibitory effect on tumor cells, which can suppress the growth of tumor cells, and lead to tumor nucleus pyknosis. Its mechanism may be related with inducing tumor cell apoptosis, blocking tumor cell at the cell cycle, suppressing oncogene expression, inhibiting tumor angiogenesis and eliciting immune reaction against tumor cells.^6–8^ Studies have verified that salidroside could inhibit the growth of a variety of cancers, but its mechanism against gastric cancer cells has not been fully clarified.^9–12^

MicroRNA (miRNA) is a kind of non-encoding transcriptional regulatory small RNA molecule. It participates in cell differentiation, proliferation and survival through binding to mRNAs, leading to mRNA translation inhibition.^13–18^ Some researches have shown that miRNA abnormal transcription plays key roles in the progression of gastric cancer. MiRNA is involved in some processes such as proliferation, differentiation and apoptosis of gastric cancer cells. It is expected to become an effective biomarker for diagnosis and prognosis in gastric cancer, and is considered to be a promising therapeutic target.^19–23^

Studies have suggested that salidroside could inhibit the growth of cancers by enhancing immunity, inducing apoptosis and regulating some signal pathways.^24–26^ However, the regulation mechanism of inhibiting tumor proliferation by salidroside is still ambiguous. Based on this, the study investigated the differential expressions of miRNAs in gastric cancer cells under the treatment of salidroside, then analyzed the interaction between the differentially expressed miRNAs and its target genes, further explored the regulatory mechanism between miRNA and tumor progression.

## Materials and methods

### Salidroside solution preparation

Salidroside was purchased from Sigma (MO, USA). Salidroside was dissolved in normal saline (NS) at the concentration of 10 mmol/L. It is the stocking solution and could be diluted in NS as needed. The human gastric cancer cell line MGC803 obtained from Nanjing Bairui Biotechnology Co., Ltd. was cultured in RPMI1640 complete medium (GIBCO, USA). Rabbit anti-human MAP3K6, MMP24 and STAT3 polyclonal antibodies were purchased from Santa Cruz Biotechnology, Inc (Santa Cruz, CA, USA). EnVisionTM kits were purchased from Dako Corp (Carpinteria, CA, USA).

### Analysis of cell cycle

Gastric cancer cells were seeded in 6-well plates at a density of 3 × 10^5^ cells per well and cultivated at 37°C overnight. Then, the cells were added with 2 mg/ml (low-dose), 4 mg/ml (middle-dose) and 8 mg/ml (high-dose) salidroside for 24 h. Cells were collected and fixed with ice-cold 70% (v/v) ethanol and kept at 4°C overnight. The cell pellets were re-suspended and stained in PBS with 50 mg/ml propidium iodide (PI) and .1 mg/ml RNase I at room temperature for 30 min. Cell cycle was examined with a FACScalibur flow cytometer (BD, USA).

### Apoptosis detection

Cancer cells were dissociated with .25% trypsin and the cell density was adjusted to 1 × 10^6^/ml, then incubated with 2 mg/ml, 4 mg/ml and 8 mg/ml salidroside respectively for 24 h. Then cancer cells were collected to examine the apoptosis.

Apoptotic indices were examined for apoptotic cells. Propidium iodide (PI) and Annexin V-FITC staining (Roche Applied Science, Indianapolis, USA) were used for the apoptosis detection. 1 × 10^6^ cells of each sample were treated with RNase, then incubated with PI and Annexin V-FITC. The apoptotic cells were detected on a flow cytometer (FACSCalibur, Becton Dickinson, USA). The data from 1 × 10^6^ cells/sample were analyzed with ModFIT LT for mac V1.01 software (Becton Dickinson, USA).

### Wound healing assay

Gastric cancer cells were seeded at a density of 2 × 10^6^ cells per well in 6-well plates and cultivated at 37°C overnight. Cell monolayers were scratched with a sterile 20 μl pipette tip. Then cancer cells were treated with different concentrations of salidroside. After incubated for 24 h, the scratched areas were photographed under an Olympus microscope. Cell migration rate=(initial scratch width – scratch width after 24 hours)/initial scratch width × 100%.

### In vitro treating gastric cancer cells with salidroside

When the cancer cells reached the logarithmic growth stage, culture medium with a concentration of 4 mg/ml salidroside was added. The cells cultured with normal saline (NS) were set up as the control. The cells were collected after 24 h for further analysis.

### miRNA extraction and database construction

TRIZOL reagent (Invitrogen/Life Technologies, CA, USA) was applied to extract total RNA from samples and agarose gel electrophoresis was used to select 18–30 nt RNA fragments. The RNA fragments were analyzed by RT-PCR amplification. Agarose gel electrophoresis was used to purify the RNA band, and then the RNA database was constructed. The database was qualified by Agilent 2100 and qRT-PCR. Finally, the constructed RNA database was sequenced by Illumina Novaseq6000.

### miRNA differential expression analysis

The differential expression of miRNA was analyzed by using EdgeR software, in which the dispersion was set to .01. The screening criteria of differential miRNA was that the expression level changed more than two times compared with the control and the *p* value was less than .05 was considered to be significant.

### miRNA target gene prediction

Targetscan (version: 7.0) and Miranda (v3.3a) methods were applied for target gene prediction, and then the target genes from the two methods was taken as the result. Targetscan is to take 2–8 nt at the 5‘end of small RNA as selected sequences and the transcripts of 3’ - UTR region for prediction. The specific parameters of the Miranda software are shown as Table 1.

**Table 1.** The parameters of miranda software.

parameter	annotation
-sc 140	The score threshold is 140
-en −10	The energy threshold is −10 kcal/mol
-strict	Demand strict 5’ seed pairing
-go −4.0	The gap-open penalty is −4.0
-ge −9.0	The gap-extend penalty is −9.0

### Functional enrichment analysis of differential miRNA target genes

After searching for differential expressed miRNA genes and their mRNA target genes, KEGG pathway analysis and GO function analysis were conducted to analyze the main functions and signal pathways of these candidate genes. GO has three ontologies, revealing the molecular function, cellular component and biological process of genes, respectively. GO function analysis demonstrates not only the function classification of differentially expressed genes, but also the significance of enriched differentially expressed proteins. KEGG pathway analysis contributes to further understand the biological functions of the differentially expressed proteins. KEGG pathway enrichment analysis uses hypergeometric test to find out the pathways which are significantly enriched in differentially expressed proteins.

### Construction of miRNA target gene interaction network

To combine with the differential expressed miRNA and their target genes, the Pearson correlation coefficient between miRNA and target genes was calculated. Based on the correlation coefficient, the interaction network between differential expressed miRNA and their target genes was constructed by Cytoscape.

### Detection of the transcription of miR-1343-3p, MAP3K6, and MMP24 genes by quantitative PCR in gastric cancer cells

The expression of miR-1343-3p, MAP3K6 and MMP24 genes was examined by fluorescence quantitative PCR (Invitrogen/Life Technologies, CA, USA). The total RNA of cancer cells was extracted by Trizol method, which was reversely transcribed into cDNA, and then examined by real-time PCR. The gene fragments of miR-1343-3p, MAP3K6 and MMP24 were amplified. The PCR primers were designed by primer designer software as Table 2.

**Table 2.** The PCR primers of miR-1343-3p, MAP3K6, and MMP24 genes.

Gene	Primers sequence(5’-3’)	Production size(bp)
miR-1343-3p	F:CGCGGCCTTAATGCTAATTGTGA R:ATCCAGTGCAGGGTCCGAGG	65
MAP3K6	F:CTGGATGTTCGTTCTGGACTCACTG R:GACCGCCTCCTTCTCCACCTC	88
MMP24	F:CACTCACCATCGGAGAGGAAACAC R:GTTGAAGTTGCCGTCACAGATGTTG	114
β-actin	F:ATCATGTTTGAGACCTTCAA R:CATCTCTTGCTCGAAGTCCA	310

Fluorescence quantitative PCR was operated according to the experimental instructions. PCR amplification conditions were 94°C for 2 min, 94°C for 30 s, 56°C for 30 s, and 72°C for 1 min, a total of 30 cycles. The fluorescence intensity ratio of β-actin gene (internal reference control) was used as the relative expression of target genes.

### Detection of the expression of MAP3K6, MMP24, and STAT3 in gastric cancer cells by immunohistochemistry

SABC immunohistochemistry was applied to detect the expression of MAP3K6, MMP24, and STAT3 proteins in different groups in gastric cancer cells before and after salidroside treatment. All the cells were fixed with 4% paraformaldehyde and rinsed twice with distilled water, then incubated with 3% H_2_O_2_ for 20 min; After antigen retrieval in microwave oven with high fire for 10 min, the cells were rinsed with PBS for three times, and then incubated with normal goat serum at 37°C for 30 min. The primary antibody solution (dilution ratio: 1:100) was added to the cells at 4°C overnight, then washed with PBS for three times. After that, cells was incubated with biotinylated secondary antibody at 37°C for 45 min and washed with PBS. Then horseradish enzyme labeled streptomyces ovalbumin working solution was added to the cells at 37°C for 30 min and rinsed with PBS. The freshly prepared DAB staining was developed for 5–10 min. Human serum albumin was used as a negative control instead of the primary antibody. The positive cells showed brown or yellow staining in the cytoplasm or the nucleus. Image J image analysis software analyzed the OD value of staining cells in different experimental groups. The log ratio of OD value between staining cells and non-stained areas from 10 visual fields of each slice were analyzed under 10 × light microscope, compared with the log ratio of blank control group for further data statistical analysis.

### Statistical methods

The data are illustrated as mean ± standard deviation (SD). A one-way analysis of variance and Chi-square test between different groups were used. *p* < .05 was considered as a statistical significant difference. All the tests were conducted in duplicate and were performed three times.

## Results

### Effect of salidroside on the cell cycle arrest of gastric cancer

**Figure 1.**
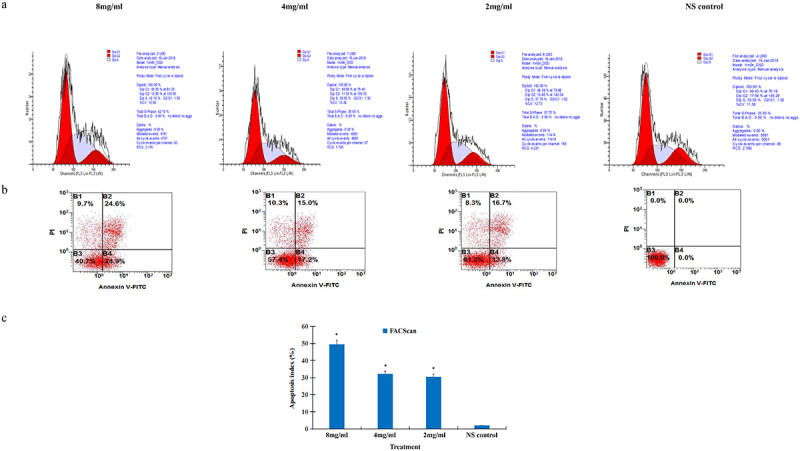
Salidroside induced cell cycle arrest and apoptosis in gastric cancer cells. The S-phase cell cycle was arrested in gastric cancer cells after salidroside treatment for 48 h (A). Gastric cancer cell apoptosis was detected by flow cytometry analysis (B). Compared with the NS control group, cancer cell apoptotic index was significantly higher in the 8 mg/ml salidroside treatment group. Chi-square test for comparison between different groups, data is presented as mean ± SD, n = 3, *p <.05 (C).

In order to determine the effect of salidroside on the cell cycle of gastric cancer cell, the cancer cells were analyzed by flow cytometry after treatment with salidroside. After cultured with different concentrations of salidroside, compared with the control group, gastric cancer cells were arrested significantly at the S-phase cell cycle. At the S-phase, the DNA level accumulated to 42.1% in the high-dose salidroside treatment, while the DNA level was 33.93% in the control group, showed in FACScan assay (Figure 1a).

### Induction of cancer cell apoptosis by salidroside

To confirm the induction of apoptosis of salidroside on gastric cancer cells, the apoptotic rates of cancer cells were detected by the FACScan assay. After culturing with different concentrations of salidroside, gastric cancer cells showed a significantly elevated apoptotic index, from 2.35 ± 1.24 to 49.50 ± 3.62% in the high-dose salidroside treatment group, compared with the NS control group (Figure 1b, *p* < .05). There was significant difference between salidroside treatment group and control group (Figure 1c).

### Salidroside inhibited the invasion of cancer cells

**Figure 2.**
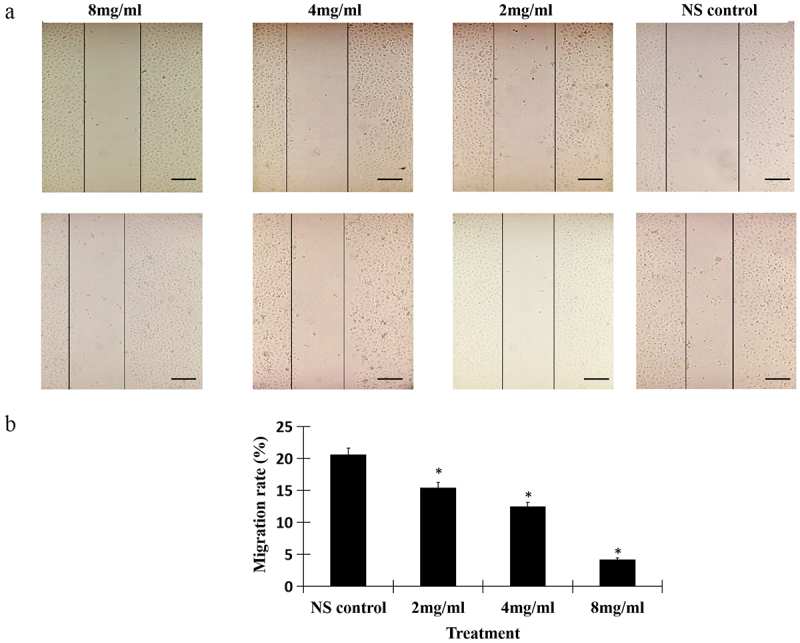
Salidroside inhibited the invasion of gastric cancer cells. Salidroside inhibited gastric cancer cells motility in a dose-dependent manner (A), and resulted in the decreased migration rates of gastric cancer cells (B). Salidroside treatment groups vs NS control group, data is presented as mean ± SD, n = 3, *p <.05. Scale bar: 50 μm.

To testify whether salidroside could suppress the migration of gastric cancer cells, we detected the invasion ability by wound healing assay. The results showed, salidroside treatment significantly resulted in the decreased wound closure in gastric cancer cells. Moreover, salidroside inhibited cancer cells migration in a dose-dependent manner (Figure 2a). In line with the wound closure, salidroside treatment resulted in the decreased migration rates of gastric cancer cells (the migration rates in the low-dose, middle-dose, high-dose salidroside treatment groups vs those from the control group, 9.89 ± 2.47, 6.03 ± 2.62, 2.53 ± 1.64 vs 14.23 ± 2.45, *p* < .05, Figure 2b). Overall, these findings suggest that salidroside exhibits an inhibitory effect on the motility of gastric cancer cells.

### Salidroside induced miRNA differential expression in gastric cancer cells

**Figure 3.**
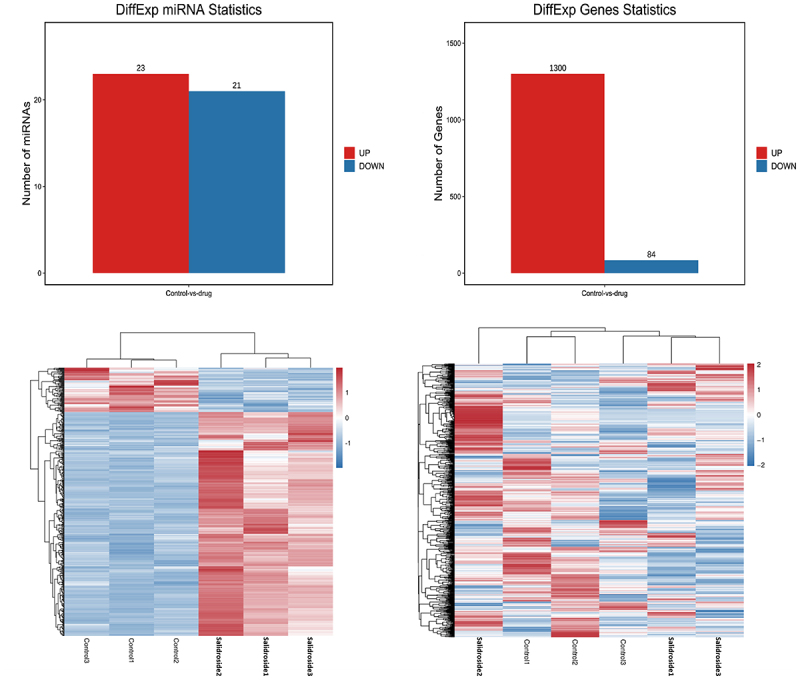
Target genes and heat map of differentially expressed miRnas. Bioinformatics analysis showed that 44 miRnas and 1384 corresponding target mRnas were expressed differentially in gastric cancer cells under the treatment of salidroside.

With the use of high-throughput sequencing, a total of 1873 miRNAs exhibited differential expression. Compared with the control group, the expression of 44 miRNAs changed significantly after salidroside treatment, *p* < .05. A total of 1384 target mRNAs were screened by these 44 miRNA target genes. Based on their differential expression levels, the miRNAs were clustered and presented as a heatmap, Figure 3.

### miRNA target gene function and pathway enrichment analysis

**Figure 4.**
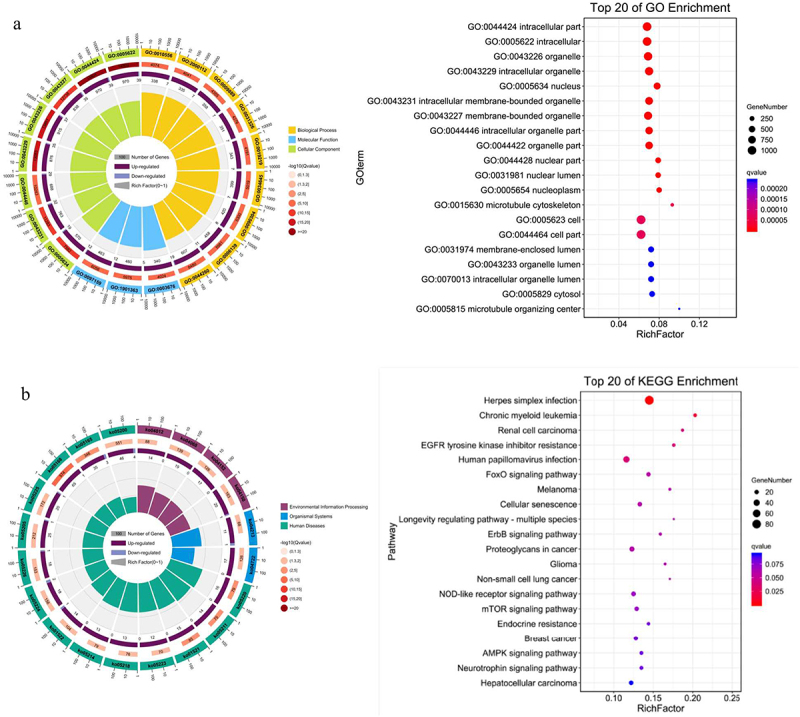
GO function and KEGG pathway analysis of miRNA target genes. GO functional enrichment (A) and KEGG pathway enrichment (B) analysis showed that the interaction between differentially expressed miRnas and their target genes was involved in most biological processes and signal pathways during the progression of gastric cancer cells, including cell proliferation, differentiation, apoptosis, metabolism, autophagy and migration. It showed that differentially expressed miRnas played important roles in regulating the proliferation of gastric cancer cells under the treatment of salidroside.

The results showed that these differentially expressed miRNAs were related with the processes of cancer cell proliferation regulation, cell hypoxia, stress response, cell migration regulation, cell metabolism, cell cycle, mitogen activated protein kinase (MAPK) cascade activation, endothelial cell proliferation regulation and blood vessel formation. Similarly, KEGG pathways relevant to differentially expressed miRNAs involved FOXO (forkhead box) signal pathway, cell cycle, hypoxia inducible factor-1α (HIF-1α) signal pathway, phosphatidylinositol 3-kinase/protein kinase B (PI3K/Akt) signal pathway, MAPK signal pathway, RAS signal pathway, IGFR signal pathway, Janus kinase (JAK)/signal transducer and activator of transcription (STAT) signal pathway (Figure 4).

GO functional enrichment and KEGG pathway enrichment analysis showed that the target genes of differentially expressed miRNA were involved in cell proliferation, invasion and metastasis. After treatment with salidroside, numerous physiological processes and signal pathways in cancer cells were significantly affected.

### Interaction between differentially expressed miRnas and target genes

**Figure 5.**
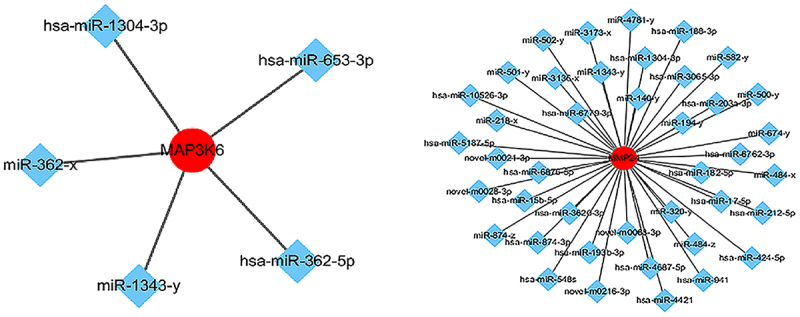
Network of miRnas and their target genes. miRNA-mRNA targeting analysis predicted that 29 mRnas were negatively regulated by miR-1343-3p, in which the mRNA genes of mitogen activated protein kinase kinase kinase 6 (MAP3K6) and membrane matrix metalloproteinase 24 (MMP24) signal pathway closely related to tumor proliferation and migration were significantly down-regulated.

In Figure 5, the red circle represents the target genes regulated by miRNA and the blue diamond represents miRNAs. Based on the analysis of the interaction network between miRNAs and target genes, specific mRNAs (e.g., mitogen activated kinase kinase kinase 6, MAP3K6, membrane matrix metalloproteinase 24, MMP24) were selected due to their interaction with miRNA and significant association with the proliferation and migration of cancer cells.

### MAP3K6 and MMP24 regulated by miR-1343-3p in gastric cancer cells

**Figure 6.**
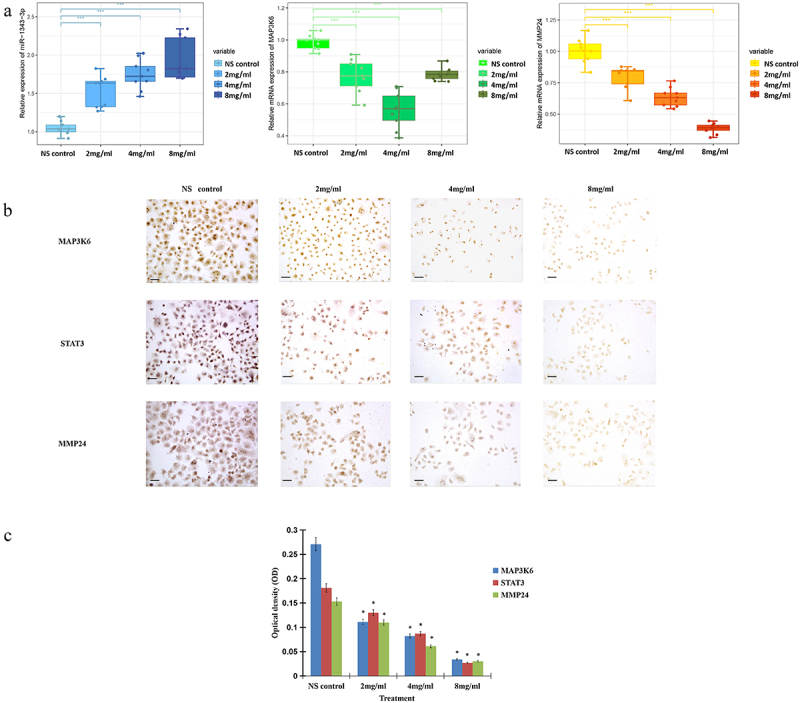
Salidroside inhibited the expression of miR-1343-3p, MAP3K6, MMP24 and STAT3 in gastric cancer cells. q-PCR results showed that compared with the NS control, the expression of MAP3K6 and MMP24 genes in gastric cancer cells were significantly down-regulated after different concentrations of salidroside treatment, whereas miR-1343-3p was significantly up-regulated. The higher the concentration of salidroside, the higher the expression level of miR-1343-3p (A). Variance analysis for comparison between different groups, data is presented as mean ± SD, n = 3, *p <.05. Immunohistochemistry showed that the expression of MAP3K6, STAT3 and MMP24 signal proteins in gastric cancer cells decreased after different concentrations of salidroside treatment. The higher the concentration of salidroside treatment, the weaker the staining of signaling molecules in the cancer cells (B) and the lower the relative ration of OD values (C). Chi-square test for comparison between different groups, data is presented as mean ± SD, n = 3, *p <.05. Scale bar: 20 μm.

In order to verify the expression of miR-1343-3p, MAP3K6, and MMP24 in gastric cancer cells after treatment with salidroside, we conducted quantitative PCR and immunostaining assay. The results showed that salidroside could significantly up-regulate the expression of miR-1343-3p, and significantly down-regulate the expression of MAP3K6 and MMP24 genes in cancer cells (Figure 6a).

Immunohistochemistry further clarified the expression of MAP3K6, MMP24, and STAT3 in gastric cancer cells after salidroside treatment. The results demonstrated that after salidroside treatment, the expression of MAP3K6, MMP24, and STAT3 genes were significantly decreased, compared with the control group. The higher the concentration of salidroside treatment, the weaker the staining of signaling molecules in the cancer cells and the lower the relative ration of OD values, *p* < .05 (Figure 6b, c).

## Discussion

It was found that salidroside could inhibit the growth of some cancers by inducing cell apoptosis, arresting cell cycle and down-regulating certain important signal pathways.^24–27^ So, we speculated that salidroside should have certain effects on gastric cancers and it is beneficial to have a deep investigation to clarify the mechanism of salidroside against tumors.

In the study, we found that salidroside suppressed the proliferation of gastric cancer cells by inducing cancer cell apoptosis, and arresting the cancer cell cycle. These data suggest that one mechanism of salidroside inhibiting the growth of gastric cancer cells is the induction of apoptosis, which is in line with the results of salidroside in other cancer researches.^26,27^

Gastric cancer is a kind of malignant tumors with high mortality. The main reason is tumor recurrence and metastasis, and the progression of tumor heterogeneity is an important factor in its development. MiRNA plays an important role in tumor cell differentiation, proliferation, apoptosis, migration and metastasis.^12–15^ The differential expression of tumor miRNA has become a hot spot and breakthrough in the current research. In this study, after salidroside treatment, the differentially expressed miRNAs were analyzed. Based on screening the miRNA target genes, GO and KEGG analysis were used to speculate the possible biological functions of these miRNAs. Combined with bioinformatics analysis, the overall interaction network between miRNAs and mRNAs was constructed. Exploration of miRNAs in gastric cancer cells with salidroside treatment would provide ideas and potential therapeutic targets for cancers.

It has been found that salidroside inhibits the growth of different malignant tumors, such as breast cancer, colon cancer, gastric cancer, and liver cancer.^24,25^ It is found that salidroside suppresses the growth of tumor cells by inducing tumor cell apoptosis and autophagy, eliciting cancer cells to differentiate into normal cells, enhancing immune reaction and blocking cancer cell cycle.^24–26^ However, the gene regulation mechanism of salidroside against tumor proliferation is still unclear.

Our recent study found that salidroside elicited the apoptosis of cancer cells and inhibited cancer cells by stimulating immune cells and releasing a number of cytokines, on the other hand, it could suppress the progression of gastric cancer by decreasing the expression of transcription factors STAT3, DEC1 and related target protein VEGF in cancer cells.^27^ In this study, 1384 target genes of miRNAs after salidroside treatment were differentially expressed in cancer cells. Most of the expression products of these target genes are closely related to tumor DNA replication, transcription, cell cycle, cell metabolism, cellular immunity, infiltration and metastasis. Salidroside inhibits the proliferation and migration of tumor cells by interfering with the expression of these products.

Studies have suggested that the abnormal expression of miRNA plays key roles in the progression of gastrointestinal cancer.^28,29^ MiRNA participates in the differentiation, proliferation and apoptosis of gastric cancer cells. Some studies have found that many miRNAs as oncogenes play important roles in the growth of gastric cancer. Therefore, using them as therapeutic targets and inhibiting their expression may be helpful to the treatment of gastric cancer. Our study showed that after salidroside treatment, there were significant differences in the expression of 44 miRNAs, including 14 newly discovered miRNAs. Bioinformatics analysis showed that there were 1384 target mRNAs correlated with differentially expressed miRNAs. The interaction between differentially expressed miRNAs and their target genes was involved in most biological processes and signal pathways during the proliferation of gastric cancer cells.

Our results showed that miR-1343-3p was silenced in the control group of gastric cancer cells, but it was induced to be highly expressed after salidroside treatment. According to the prediction of miRNA–mRNA interaction analysis, 29 mRNAs were negatively regulated by miR-1343-3p. The mRNA genes of mitogen activated protein kinase kinase kinase 6 (MAP3K6)/membrane matrix metalloproteinase 24 (MMP24) signal molecules, which are closely related to tumor proliferation and migration, were significantly down-regulated. miR-1343-3p is one of the confirmed miRNAs that can inhibit cancer growth in recent years, but it is expressed lowly in a variety of solid tumors.^30–32^ Our results showed that MAP3K6 and MMP24 were the target genes of miR-1343-3p. miR-1343-3p binds the target mRNA genes MAP3K6 and MMP24 in gastric cancer cells, and then suppresses the expression of MAP3K6 and MMP24, inhibiting the proliferation and invasion of cancer cells.

Although the role of MAP3K6 in gastric cancer is unclear, the function of MAP3K6 is likely to be correlated with the development of gastric cancer. During the progression of gastric cancer, many signal transduction pathways, such as MAPK, JAK-STAT, MMP, RAS, PI3K/Akt, and other pathways, are activated successively.^33–35^ MAP3K6 interacts with STAT3 gene and enhances its expression. These signal transduction pathways probably take part in the progression of gastric cancer. Our results showed that salidroside significantly down-regulated the expression of MAP3K6 and STAT3 genes in gastric cancer cells, indicating that salidroside suppressed the proliferation and progression of gastric cancer cells by inhibiting the expression of these pathway molecules.

Tumor migration are closely correlated with extracellular matrix (ECM). MMPs can degrade basement membrane and ECM, which play a key role in tissue reconstruction. This process is mainly depended on extracellular matrix metalloproteinases (MMPs).^34,35^ Overexpressed MMPs often degrade basement membrane and ECM, then promote tumor angiogenesis and invasion.^36,37^ MMP24 is a subfamily of matrix metalloproteinases. It is a new member of the membrane type matrix metalloproteinase (MT-MMPs) family. MMP-24 was detected in malignant glioma, breast cancer and ovarian cancer.^38,39^ Studies have showed that MMP24 mRNA was overexpressed in gastric cancer, which was probably related with tumor metastasis.^40^ Based on our results, it is presumed that salidroside may inhibit the proliferation and invasion of gastric cancer cells by down-regulating MAP3K6/MMP24 signaling molecules through up-regulating miR-1343-3p.

Studies have shown that miR-1343-3p is a short non-encoding RNA (20-24nt), which participates in the regulation of mRNAs by influencing the stability and translation of mRNAs.^41,42^ Studies have shown that miR-1343-3p is down-regulated in pancreatic cancer, prostate cancer and colon cancer. Abnormal transcription of miR-1343-3p can affect the growth and migration of tumors.^42–44^ miR-1343-3p in lung cancer is inhibited by lncRNA 02323, resulting in up-regulated expression of TGFBR1 and promoting interstitial transformation and cancer cell metastasis.^43,44^ The expression and activation of gastric cancer carcinogen TEAD4 are also silenced by miR-1343-3p.^45^ However, the regulatory relationship between miR-1343-3p, MAP3K6 and MMP24 in gastric cancer cells has not been reported. We found that the MAP3K6 and MMP24 signal molecules were regulated by miR-1343-3p in gastric cancer cells, and miR-1343-3p was negatively correlated with the expression of these signal molecules.

These studies suggest that miR-1343-3p, as a tumor suppressor, is silenced after being bound by lncRNA, losing the ability to inhibit downstream mRNA target genes, which result in cancer cell proliferation and migration. Therefore, inducing or promoting miR-1343-3p expression is likely to be a therapeutic strategy to inhibit tumor proliferation and migration. Our experimental results showed that salidroside promoted the expression of miR-1343-3p in gastric cancer cells and down-regulated the expression of MAP3K6 and MMP24 mRNA genes significantly. Therefore, it is suggested that salidroside may inhibit the proliferation and invasion of gastric cancer cells by increasing the expression of miR-1343-3p and decreasing the expression of target mRNA genes. So, it is of great significance to further study the interaction network between differentially expressed miRNAs and target mRNA genes regulated by salidroside. These differentially expressed miRNAs and their target genes may become important regulatory factors and potential therapeutic targets for cancers.

## Conclusions

To sum up, this study is the first report to prove the effect of salidroside on tumor suppressor miRNA-1343-3p in gastric cancer. The study suggested that salidroside inhibited the growth of gastric cancer by inducing cancer cell apoptosis, arresting cell cycle and down-regulating the related signal transduction pathways. After treatment with salidroside, miRNAs play important roles in regulating proliferation and metastasis in cancer cells. Salidroside is likely to suppress the growth of gastric cancer by up-regulating the tumor suppressor miR-1343-3p and down-regulating the expression of MAP3K6 and MMP24 signal molecules. Therefore, to fully comprehend the potential effects of salidroside, it is necessary to further explore the interaction network between the differentially expressed miRNAs and related targeted genes, which may become potential therapeutic targets for cancer treatment.

## Data Availability

The datasets analyzed in the present study are available from the corresponding author on reasonable request.

## Abbreviations

miRNA: microRNA
MAP3K6: mitogen activated protein kinase kinase kinase 6
MMP24: membrane matrix metalloproteinase 24
STAT3: signal transducer and activator of transcription 3
NS: normal saline
PI: propidium iodide
FITC: fluorescein isothiocyanate
KEGG: Kyoto encyclopedia of genes and genomes
GO: gene ontology
DAB: diaminobezidin
OD: optical density
AI: apoptotic index
FOXO: forkhead box
HIF-1α: hypoxia inducible factor-1α
PI3K/Akt: phosphatidylinositol 3-kinase/protein kinase B
IGFR: insulin growth factor receptor
JAK: Janus kinase
DEC1: differentiated embryo-ehondrocyte expressed gene 1
VEGF: vascular endothlial growth factor
ECM: extracellular matrix
MMPs: matrix metalloproteinases
TEAD4: transcriptional enhanced associate domain4
